# The influence of an external magnetic field on the dynamics of magnetite reduction with hydrogen

**DOI:** 10.1039/d1ra01200b

**Published:** 2021-04-26

**Authors:** Petr A. Chernavsky, Nellie V. Kim, Victor A. Andrianov, Yurii D. Perfiliev, Alla A. Novakova, Nikolai S. Perov

**Affiliations:** Department of Chemistry, Lomonosov Moscow State University 1-3 Leninskie Gory Moscow 119991 Russia; Faculty of Physics, Lomonosov Moscow State University 1-2 Leninskie Gory Moscow 119991 Russia perov@magn.ru perov@physics.msu.ru; Scobeltsyn Institute of Nuclear Physics, Lomonosov Moscow State University 1-2 Leninskie Gory Moscow 119991 Russia

## Abstract

The kinetics of hydrogen reduction of magnetite was investigated in different magnetic fields. The magnetic moment measurements *in situ* were used for the control of the reaction. A strong difference in the magnetic properties of the reaction results was obtained for applied strong and weak magnetic fields. The X-ray diffraction and Mössbauer spectra of the reduced samples confirmed their different composition. The mechanism of the magnetic field effect is discussed.

## Abstract 2

The kinetics of hydrogen reduction of magnetite was investigated in different magnetic fields.

## Graphical abstract

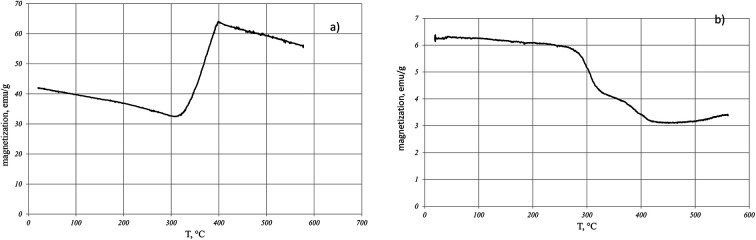

## 1 Introduction

Any chemical process in which carbon can be replaced by molecular hydrogen H_2_ as a reducing agent is potentially important for solving the problem of global CO_2_ emissions. For example, in the metallurgical industry, 1.8 tons of CO_2_ are accounted for each ton of produced steel, and this gas comprises 8% of global CO_2_ emissions.^1^ The use of hydrogen for the direct reduction of iron ore makes it possible to obtain steel with a low carbon content, that significantly improves its quality.^2^ At atmospheric pressure and *T* > 450 °C, the reduction process includes three stages (hematite–magnetite–wustite–iron), while at lower temperatures wustite (FeO) does not form.^3^ The experimentally it is confirmed the presence of the thermodynamically unstable FeO oxide as an intermediate recovery product is relatively rarely mentioned in the literature. The wustite formation was observed during the reduction of magnetite at temperatures above 500 °C^4^ Metastable FeO is formed in iron-supported catalysts due to the stabilizing action of carriers (MgO, SiO_2_, Al_2_O_3_). The carrier surface stabilizes the wustite formation due to the strong oxide–oxide interaction.^5^ The formation of FeO at *T* > 570 °C as an intermediate in the reduction of magnetite with hydrogen, regardless of kinetic or diffusion limitations, was confirmed by the authors of.^6^ In the temperature range 350–570 °C, instead of direct reduction of magnetite to iron, a two-stage process on the surface of magnetite is possible, at least on an atomic scale. It is assumed that in the reversible disproportionation reaction, 4FeO = Fe_3_O_4_ + Fe, FeO is formed at the interface between Fe_3_O_4_ and Fe.^6^ The effects of the magnetic field on the reduction reactions of metal oxides were investigated in a number of works^7,8^ mainly devoted to the reduction reaction of hematite. However, the reliability of the obtained experimental data and the lack of explanation of the nature of the effect should be noted.

The hypothesis explaining the influence of the magnetic field on topochemical reactions was put forward by Bulgarian researchers – G. P. Visokov and D. G Ivanov^9^ and is based on the assumption of a change in the magnetic moments of the starting materials and products during the chemical reaction. The positive effect of applying magnetic fields on the heat conduction, reaction kinetics, and hydrogenation time of a lanthanum nickel bed was presented in.^10^ A large amount of experimental data was obtained by a group of researchers led by Rowe.^11,12^ They investigated the effect of the external magnetic field (4̄200 Oe) on the reduction reactions of iron, cobalt and nickel oxides. The authors used the thermogravimetric method. In a number of studies, saturation magnetization was additionally measured, from which the composition of the reaction mixture was calculated. The difference in the values obtained by those two methods, on average, did not exceed several percent. The authors did not find an explanation of the observed phenomenon nature. Subsequently, the results of these studies were called into question.^13^

It should be noted that in none of the cited works the method of direct control of the magnetization of the reaction mixture during the reaction was not used. The influence of an external magnetic field on the kinetics of magnetite reduction has not been studied. In the present work, an attempt was made for the first time to study the effect of an external magnetic field on the kinetics of hydrogen reduction of magnetite.

## 2 Experimental section

Magnetite (Fe_3_O_4_) nanopowder, 50–100 nm particle size, 97% trace metals basis Sigma-Aldrich was used as the object of study. The experiment was carried out on a vibration sample magnetometer in which a flowing quartz microreactor served as a magnetic sample.^14^ The magnetic moment calibration was made with a pure cobalt bulk sample of 10 mg. The mass of the magnetite sample was 10 mg in all experiments. Before kinetics studies, magnetite samples were calcined with Ar flow 10 cm^3^ s^−1^ at linear heating of 10 °C min^−1^ to the temperature of 600 °C in the external magnetic field of 60 Oe. For further research, hydrogen (99.993%) was used without preliminary purification. Isothermal experiments were carried out in the temperature range 340–450 °C. The external magnetic field was varied in the range from 60 Oe to 5000 Oe with the external electromagnet. The saturation magnetization was measured at the magnetic field from of 7000 to 8000 Oe by extrapolation to the zero field.

Diffraction patterns were recorded on a PANalytical Empyrean diffractometer in Bragg–Brentano geometry (mode – 2 scans, 40 mA, 40 kV) in increments of 0.026 degrees, in the range of angles from 5 to 100 degrees using a Pixel3D detector. The anode material is Cu. Diffraction patterns were analyzed using HighScore Plus PANalytical software with full-profile analysis. Magnetite samples for research were prepared according to the method described above. After cooling in hydrogen, the samples were passivated in a stream of technical Ar, and then transferred to a diffractometer.

Mössbauer spectroscopy measurements were carried out on a standard spectrometer with constant acceleration in the transmission geometry with a ^57^Co source in Rh. The Mössbauer spectra were decomposed into components using the least squares method using the program “UnivemMS”.

## 3 Results and discussion

**Fig. 1:**
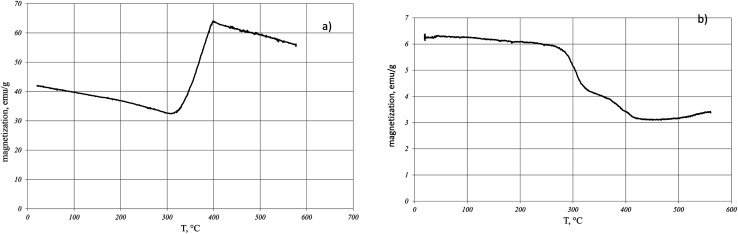
Temperature dependence of magnetization during the reduction of magnetite in hydrogen in a field of 5000 Oe (a) and in a field of 60 Oe (b).

Fig. 1 shows the dependence of the magnetization *J*(*T*) on temperature during the reduction of magnetite in hydrogen for two values of the external magnetic field.

**Fig. 2:**
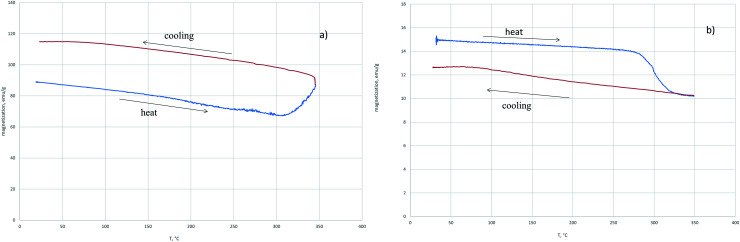
The dependence of magnetization on temperature in the heating–cooling mode on the value of the external field 5000 Oe (a) and 60 Oe (b) in the hydrogen flow.

In the field of 5000 Oe at the temperature of 310 °C, an increase in magnetization is observed which at *T* = 400 °C is replaced by the magnetization decrease (Fig. 1a). In contrast, in the external field of 60 Oe (Fig. 1b), a nonmonotonic decrease in the magnetization is observed with temperature increasing. The saturation magnetization after cooling the sample reduced in the 5000 Oe field showed that magnetite was completely reduced to iron. At the same time, a similar procedure performed with the sample of magnetite reduced in the 60 Oe field indicates a partial reduction of magnetite. When the sample reducing in H_2_ was heated to the temperature of 350 °C and then cooled, the magnetization after cooling was greater (Fig. 2a) or less (Fig. 2b) than the initial magnetization before. The reduction process depends on the applied external magnetic field.

**Fig. 3:**
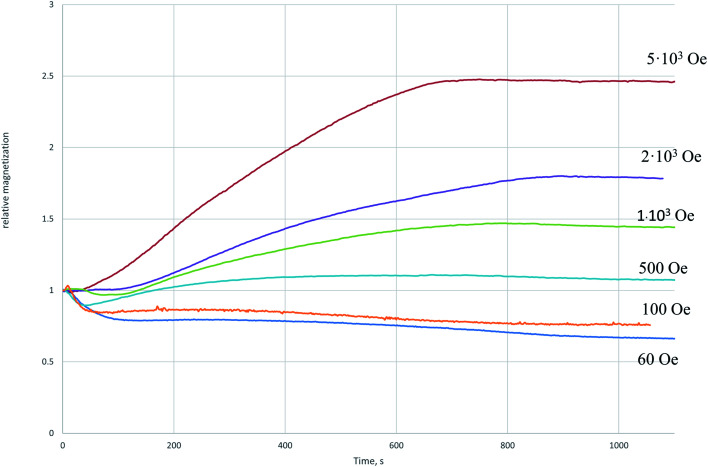
The dependence of the relative magnetization on the recovery time at 350 °C in the different magnetic field. The curves were normalized to the initial magnetic moment.

The relative increase in the magnetization after cooling of the sample restored in the field of 5000 Oe (Fig. 2a) indicates the appearance of a phase in the system with a higher specific magnetization than the initial magnetite. The decrease in the magnetization with respect to the initial one (Fig. 2b) indicates the transition of part of magnetite to the weak magnetic phase. In Fig. 3 the dependences of the relative magnetization (*J*/*J*_0_ where *J*_0_ is the initial value of magnetization) on time of isothermal reduction at *T* = 350 °C are shown in the different magnetic fields. From the data presented it follows that in the magnetic field being stronger than 500 Oe the magnetization increase is observed with time.

**Fig. 4:**
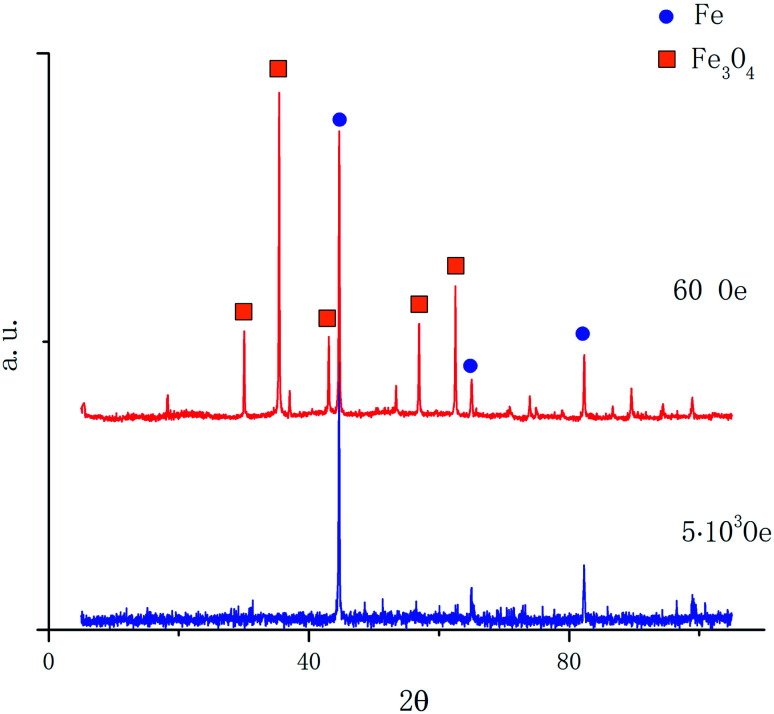
Diffraction patterns of magnetite samples reduced in a field of 60 Oe and 5000 Oe at a temperature of 450 °C.

To analyze the composition of the reduced sample the X-ray diffraction was used. In Fig. 4 the X-ray diffraction patterns of magnetite samples reduced in H_2_ for 60 minutes are presented for magnetic fields of 60 Oe and 5000 Oe at the temperature of 450 °C. After reduction, the samples were passivated in the technical Ar stream 10 cm^3^ s^−1^ at the room temperature during 15 min. This procedure allowed us to avoid oxidation of iron nanoparticles in the process of obtaining diffraction patterns.

From the data presented in Fig. 4, it follows that the iron concentration in the sample reduced in the magnetic field of 5000 Oe is completely composed of iron. Quantitative phase analysis of the diffraction pattern of the sample reduced in the magnetic field of 60 Oe consists of 70% magnetite and only 30% of iron.

**Fig. 5:**
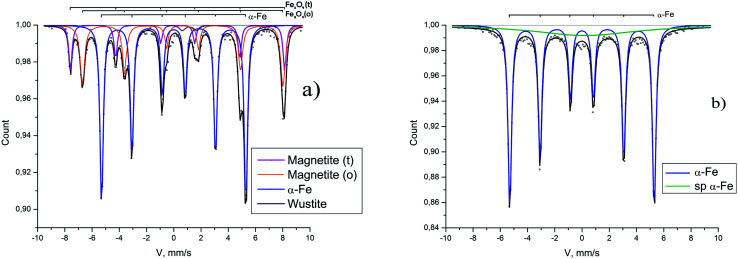
Mössbauer spectra on ^57^Fe nuclei. (a) The sample was prepared in magnetic field 60 Oe. α-Fe *H*_hf_ = 330 kOe *P* = 58%. Fe_3_O_4_*H*_1hf_ = 488 kOe, *H*_2hf_ = 458 kOe *P* = 40%. (b) The sample was prepared in the magnetic field 5000 Oe. α-Fe *H*_hf_ = 330 kOe *P* = 84%.

Similarly prepared reduced samples were studied with Mössbauer spectroscopy: the Mössbauer spectra of the samples reduced in the magnetic field of 60 Oe and 5000 Oe are presented in Fig. 5.

The common component of two spectra is a sextet with hyperfine magnetic field about 330 kOe. It corresponds to the metallic α-iron.^15^ The spectrum of the sample reduced in the field of 60 Oe is shown in Fig. 5a where two more sextets are visible, the parameters of which (the hyperfine magnetic fields 4884 and 4569 kOe) characterize the magnetite^16^ remaining due to incomplete reduction. Besides there is a small doublet component (2%) corresponding to wustite.^17^ It follows from the Mössbauer data that 58% of magnetite is reduced in low field of 60 Oe to α-iron (see Table 1).

**Table 1:** Mössbauer spectra parameters^a^

*H*_0_	Phase	*H*_hf_, kOe, ±1	*Δ*, mm s^−1^, ±0.1	*δ*, mm s^−1^, ±0.1	*A*, %, ±1	*Γ*, mm s^−1^, ±0.01
60 Oe	Magnetite (t)	488	0.0	0.3	14	0.27
	Magnetite (o)	457	0.0	0.6	26	0.39
	α-Fe	330	0.0	0.0	58	0.29
	Wustite	—	1.0	1.1	2	0.50
5000 Oe	α-Fe	330	0.0	0.0	84	0.29
	Sp α-Fe	—	1.2	0.0	16	7.80

a Notes: isomer shift (*δ*). Quadrupole splitting (*Δ*). Magnetic hyperfine field (*H*_hf_). Full line width at half maximum (*Γ*). Relative areas of spectral components (*A*) represent relative contents of the corresponding Fe forms assuming a common recoilless fraction for all forms in a sample.

The spectrum of the sample obtained in the field of 5000 Oe is shown in Fig.5b. It contains the main component of metallic α-iron with 84% intensity, and the relaxation component with very large line width, the appearance of which is associated with ultra-small superparamagnetic iron particles (less than 10 nm) and with traces of magnetite. Thus, in a magnetic field of 5000 Oe, more than 84% of magnetite is reduced to α-Fe.

From the above presented X-ray diffraction and Mössbauer spectroscopy data, it follows that the reduction of magnetite in the magnetic field of 60 Oe and in the field of 5000 Oe leads to significantly different results. In our opinion, the decrease in magnetization upon reduction in a 60 Oe field is due to the formation of the antiferromagnetic phase of wustite FeO as a result of the topochemical process

1 Fe_3_O_4_ + H_2_ = 3FeO + H_2_O

The non-monotonic character of the decrease in magnetization (Fig. 1b) in the temperature range from 300 °C to 400 °C indicates the multi-stage recovery process and the existence of several non-stoichiometric FeO_1−*x*_ phases (where *x* < 1) differing in total magnetic moments. One of the reasons for the nonmonotonic change in magnetization can be the disproportionation reaction of wustite:

2 4FeO = Fe_3_O_4_ + Fe.

The detailed mechanism of the process, apparently, includes the interaction of surface oxygen (O)_s_ with hydrogen H_2_ followed by the formation of an anionic vacancy (V)_s_(O)_s_ + H_2_ = (V)_s_ + H_2_O.

A fundamentally different situation is observed in the field of 5000 Oe (Fig. 1a). In this case, at *T* ≥ 310 °C, a monotonic increase in magnetization occurs up to 400 °C. At 400 °C, the increase in magnetization is replaced by a decrease, which indicates the completion of the recovery process, and a further change in the magnetization is due to the temperature dependence of the iron magnetization near the Curie temperature. Measurement of the saturation magnetization of the sample after cooling indicates a complete reduction of magnetite to iron. The increase in magnetization at *T* ≥ 310 °C indicates the predominant role of a one-stage process

3 Fe_3_O_4_ + 4H_2_ = 3Fe + 4H_2_O

Thus, an external magnetic field changes the mechanism of the recovery process and already in a field exceeding 1000 Oe a noticeable contribution of reaction (3) and a decrease in the contribution of reaction (1) are observed. In all cases, the process includes a nucleation stage. Nucleation, in turn, suggests the presence of active centers on the surface. The comprehensive theoretical model and also experimental study describing the action of an external magnetic field on a chemical process in a heterogeneous reaction on solid has not been clearly reported. We suggest that an external magnetic field may affect the concentration of nucleation centers. The work^18^ shows that spin-structure can strongly affects orbital occupation of the surface and especially charge-transfer between the solid surface and the adsorbate.

## 4 Conclusion

The magnetic moment sensitive technique was used to investigate the iron oxides reduction by hydrogen in the magnetic field. It is shown that H_2_ adsorption and dissociation are modified by changes in spin-structure. Thus, the external magnetic field can affect the rate of a topochemical reaction.

## Author contributions

PC – purpose of investigation and chemical reactions, data analysis, writing the manuscript, discussion the text; NK – X-ray diffraction, data analysis and corresponding description, discussion the text; VA – Mössbauer measurements and analysis, corresponding description, discussion the text; YP – Mössbauer data analysis; AN – X-ray diffraction and Mössbauer data analysis, discussion the text; NP – magnetic properties analysis, manuscript preparation, discussion the text.

## Conflicts of interest

There are no conflicts to declare.

## Supplementary Material
